# Robotic cholecystectomy during surgical training: learning curve and perioperative outcomes of 441 consecutive cases in a high-volume teaching center

**DOI:** 10.1007/s11701-026-03558-4

**Published:** 2026-06-22

**Authors:** Gianpiero Gravante, Maria Teresa Mita, Amedeo Altamura, Vittoria Barbieri, Gianfranco Bianco, Sara Benedicenti, Alberto Catamerò, Lavinia Clemente, Gloria Giaracuni, Giorgia Grillea, Barbara Leone, Giulia Morelli, Arcangelo Picciariello, Giuliana Puglisi, Roberto Sorge, Francesco Rubichi, Massimo Giuseppe Viola

**Affiliations:** 1 Department of General Surgery, Azienda Sanitaria Locale ASL Lecce, Casarano, Italy; 2 Department of General, Robotic and Oncologic Surgery, G. Panico Hospital, Tricase, Lecce, Italy; 3 Università Campus Bio-Medico di Roma, Roma, Italy; 4 Università degli studi di Bari Aldo Moro, Bari, Italy; 5 Department of General Surgery, Azienda Sanitaria Locale ASL Lecce, Scorrano, Italy; 6 Department of Experimental Medicine, University of Salento, Lecce, Italy; 7 Department of Human Physiology, Laboratory of Biometry, University of Tor Vergata, Rome, Italy

**Keywords:** Robotic surgery, Surgical education, Robotic training program, Learning curve, Minimally invasive surgery, Laparoscopic cholecystectomy, Gallbladder disease, Perioperative outcomes

## Abstract

Robotic cholecystectomy (RC) has been increasingly adopted in general surgery; however, evidence on its role and performance within structured training programs remains limited. This study aimed to evaluate the learning curve and perioperative outcomes of RC in a high-volume teaching center. A single-center retrospective study was conducted in a high-volume teaching hospital including all consecutive RCs performed between October 2019 and April 2025 by surgeons and trainees without prior robotic experience. All procedures were carried out within a structured training program under senior supervision. The primary outcome was the learning curve, assessed using cumulative sum (CUSUM) analysis of operative time. Secondary outcomes included perioperative results and subgroup analyses according to operator type and patient-related factors. Multivariable regression analysis was performed to identify factors influencing operative time. A total of 441 patients were included. Mean operative time was 64 ± 21 min. No intraoperative injuries or conversions to open or laparoscopic surgery occurred. Postoperative complications were observed in 6 patients (1.4%), with a readmission rate of 0.9%. CUSUM analysis identified four phases: an initial variability phase (1–13 cases), a stabilization phase (14–30), a performance improvement phase (30–50), and a late plateau. Operative time was independently associated with adhesiolysis (B = 9.382; *p* < 0.001) and male sex (B = 7.139; *p* < 0.001), whereas operator type was not significant. Outcomes were consistent across subgroups, including obesity and ASA class. Comparable learning patterns were observed between surgeons and trainees. In high volume training centers, RC can be safely implemented within surgical training programs, showing a relatively short learning curve and low complication rates. These findings support its use as a reproducible platform for robotic training and suggest its suitability as an entry-level procedure in robotic surgery.

## Introduction

Laparoscopic cholecystectomy (LC) remains the gold standard for the treatment of benign gallbladder disease, offering low morbidity, short hospital stays, and rapid recovery. Over the past decade, robotic cholecystectomy (RC) has progressively gained adoption, driven by enhanced three-dimensional visualization, wristed instrumentation, improved ergonomics, and greater precision during dissection [1, 2] which has been associated in some studies with reduced odds of bile duct injury and conversion to open when compared to LC [3]. Furthermore, given its high procedural volume, widespread adoption, and standardized operative steps, RC represents an ideal index procedure for surgical training, allowing surgeons to safely familiarize themselves with the robotic platform and its technical specificities [4].

Assessing outcomes in a training environment is essential to determine whether RC can be safely adopted without compromising patient outcomes, while simultaneously facilitating surgical education. Limited real-world data specifically address the integration of RC within structured surgical training programs. Most available evidence derives from expert-led series [4], small institutional cohorts [5–7], or limited comparative studies between LC vs. RC [8, 9], leaving uncertainty regarding its safety and reproducibility in high-volume teaching settings. Furthermore, the performance of RC in clinically challenging scenarios - such as obese patients, frail patients, emergency procedures - has not been consistently evaluated. These subgroups are of particular relevance, as they represent situations in which technical advantages of the robotic platform may translate into measurable clinical benefit. Large-volume experiences may provide valuable insight into safety, feasibility, and reproducibility across varying degrees of operative complexity.

The aim of this study was to evaluate the learning curve and perioperative outcomes of 441 consecutive RC performed in a high-volume university-affiliated hospital within a structured surgical training program, with predefined subgroup analyses according to obesity and clinically relevant subgroups (sex, ASA, adhesiolysis).

## Materials and methods

### Study Design

The study is a single-center retrospective study conducted at a high-volume university-affiliated hospital and has been reported according to the Strengthening the Reporting of Observational Studies in Epidemiology (STROBE) guideline [10].

All RC procedures performed between October 2019 and April 2025 by operators (surgical trainees and surgeons) without prior robotic experience were retrospectively analyzed and included in this study. All RC cases were supervised by a senior surgeon with extensive advanced laparoscopic experience. RC performed by operators who already had robotic experience, or those patients undergoing LC and open cholecystectomy were excluded. A prospectively maintained database was used to select patients. Informed consent was obtained from all individual participants included in the study.

### Training protocol

Our robotic training program begins with the Intuitive Surgical preclinical training course (TR100, Technology Training), which focuses on the fundamental technical aspects of the da Vinci Surgical System. Following completion of TR100, each surgeon is required to undertake a minimum of 20 h of robotic simulation training, achieving a target average score above 90, and to participate as a bedside assistant in at least 10 procedures of moderate-to-high complexity (cholecystectomies, colectomies). Subsequently, surgeons progress to supervised procedural training, during which they begin performing robotic cholecystectomies as primary operators. The structured robotic training pathway was initially implemented for junior attending surgeons without previous robotic experience. After completion of the same standardized curriculum, surgical trainees subsequently entered the program and underwent an identical training process, including theoretical instruction, simulation training, bedside assisting, and supervised console activity. The training pathway requires completion of at least 20 robotic cholecystectomies, with the first 10 cases performed under direct supervision of an experienced surgeon. Upon completion of this phase and the advanced procedural training module (TR300, Surgeon-Led Procedure Training), surgeons are allowed to progress to independent console practice in robotic cholecystectomy. Further progression includes transition to more complex procedures, such as colorectal surgery.

The attending surgeons included in the robotic training pathway had already completed the learning curve for laparoscopic cholecystectomy and had variable experience in laparoscopic colorectal surgery as primary operators, while also participating as assistants in major minimally invasive procedures. Surgical trainees were not senior-level residents and entered the robotic program only after prior institutional exposure to minimally invasive surgery and completion of the same structured robotic curriculum. During the study period, participating surgeons were exclusively engaged in robotic cholecystectomy, with no concurrent robotic surgical procedures performed.

### Robotic procedure

**Fig. 1:**
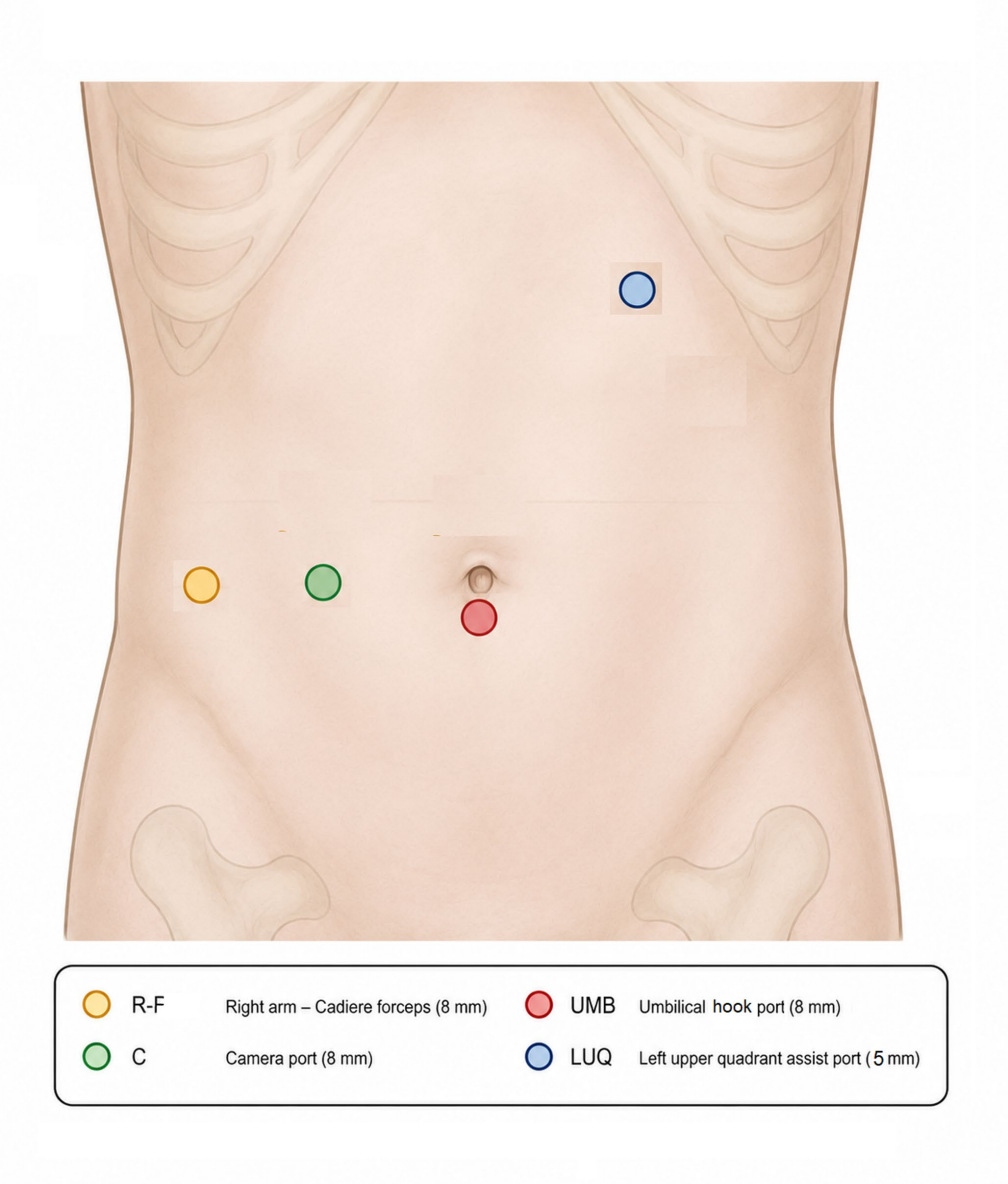
Robotic port placement for cholecystectomy. R-F: right flank, C: camera, UMB: umbilical, LUQ: left upper quadrant

All procedures were performed using the da Vinci Xi^®^ Surgical System (Intuitive Surgical, Sunnyvale, CA, USA). Standard antibiotic and antithrombosis prophylaxis were administered to all patients. Using an open technique for first trocar entry, an 8-mm robotic trocar was inserted at the umbilicus, and pneumoperitoneum was established at 12 mmHg. Two additional 8-mm trocars were placed under direct vision along the right midclavicular line and in the right flank, at the level of the umbilicus. An additional 5-mm assistant port was inserted in the left upper quadrant (Fig. 1). The da Vinci system was docked by bringing it over the patients’ right shoulders. The umbilical port was used for the cautery hook, the right midclavicular port for the camera (8 mm Endoscope Plus, 30° da Vinci X/Xi Surgical System), the right flank port for the Cadiere forceps (Fig. 1). The accessory port was used for the cephalad traction of the fundus of the gallbladder, and dissection of the Calot triangle commenced to obtain the critical view of safety. Once the critical view was obtained, the cystic duct and artery were closed with Hem-O-Lock (one proximally, two distally). They were then divided, and the gallbladder was dissected from the gallbladder fossa using cautery. The specimen was inserted in an Endocatch bag and removed it through the umbilical port. Finally, the port sites and skin were closed.

Demographics and clinical data were recorded for each patient including age, sex, Body Mass Index (BMI) and American Society of Anesthesiologists (ASA) class, indication for operation, and prior abdominal surgeries. The type of operator (surgeons or surgical trainees), total operative time (the time from skin incision to closure, including robotic docking and undocking time), conversion to open or laparoscopic surgery, intra- and postoperative complications, length of hospital stay and readmissions were all recorded. Obesity was defined as patients with BMI greater than 30. Postoperative complications were determined using the Clavien-Dindo classification [11].

### Outcomes

The primary outcome of this retrospective series was to assess the RC learning curve, using operative time as a surrogate marker. A subgroup analysis was performed according to operator type (surgeons vs. surgical trainees). Secondary outcomes included safety parameters, such as postoperative complications, conversion to open or laparoscopic surgery, and readmission rates. Additional subgroup analyses were conducted based on sex, obesity status, ASA grade, previous abdominal surgery, and the need for adhesiolysis.

### Statistical analysis

All data were inserted into an Excel database (Microsoft, Redmond, Washington – United States) and analyzed with the Statistical Package for the Social Sciences Windows version 27.0 (SPSS, Chicago, Illinois, USA). Descriptive statistics used were the mean ± standard deviation for continuous parametric variables, median and interquartile range (IQR) for non-parametric variables, and frequencies for categorical variables. Normality assumptions were demonstrated with histograms and the Shapiro–Wilk test.

The cumulative sum (CUSUM) analysis was performed using Microsoft Excel for Windows 11 (Microsoft Corp., Redmond, WA, USA). Operative times from all procedures (*n* = 441) were used to construct the CUSUM curve, taking the overall mean operative time as reference. Positive and negative cumulative deviations from the overall mean operative time were calculated and plotted separately according to the practical methodology described in the QI Macros CUSUM guide. The analysis was performed on the pooled chronological case sequence, reflecting program-level performance rather than individual learning curves [12]. The point of inflection of the curve was identified to estimate the number of procedures required to achieve procedural stabilization and operative proficiency. The phases identified in the CUSUM analysis were primarily determined through visual inspection of the curve morphology and changes in slope; no formal statistical change-point analysis was performed.

Comparisons between subgroups were performed using ANOVA one-way for continuous parametric variables, and the Mann–Whitney U test or Kruskal–Wallis test for non-parametric variables, as appropriate. Categorical variables were analyzed using the Chi-square test or Fisher’s exact test when expected cell counts were less than 5. The relationship between operative time and case sequence was initially explored using Spearman’s rank correlation.

A regression analysis of operative time was performed including variables that were significant at univariate analysis (method “enter”). A *p*-value < 0.05 was considered statistically significant.

## Results

Between October 2019 and April 2025, a total of 457 patients underwent RC performed by 22 operators (10 surgeons and 12 trainees). Sixteen patients were excluded due to incomplete data, leaving 441 patients available for the final analysis. All trainees’ cases included in the study were entirely performed by the resident surgeon, without any co-surgery or hybrid cases.

### Demographics and clinical characteristics

The mean age at the time of surgery was 58 ± 13 years, and 159 patients were male (36.1%). A total of 156 patients (35.4%) were classified as obese (BMI ≥ 30). The ASA physical status was grade I in 160 patients (36.3%), grade II in 243 (55.1%), and grade III in 38 patients (8.6%). The majority of patients presented with biliary colic (*n* = 313, 71.0%); previous episodes of cholecystitis were reported in 28 patients (6.3%). Four patients (0.9%) had concomitant choledocholithiasis, including two cases of cholangitis (0.5%); seven patients (1.6%) presented with biliary pancreatitis. The most frequent indication for surgery was cholelithiasis (*n* = 379, 85.9%), followed by adenomyomatosis (*n* = 47, 10.7%), or both (*n* = 14, 3.2%). One patient with cirrhosis underwent RC due to suspicious gallbladder wall thickening.

Mean operative time was 64 ± 21 min. Adhesiolysis was performed in 144 patients (32.7%); 63 patients (14.3%) received an abdominal drain, which was removed on postoperative day 1 in all but two cases, where removal occurred on day 2. No intraoperative iatrogenic injuries were recorded, and no conversions to open or laparoscopic surgery were required. Six patients (1.4%) experienced postoperative complications: two complications occurred within 30 days, and four beyond 30 days. Three patients had Clavien-Dindo grade 2 complications, three patients grade 3. Early postoperative complications consisted of a postoperative bleeding from a trocar, controlled the same day of surgery with a laparoscopic approach (Clavien-Dindo grade 3), and one patient who experienced postoperative fever on the first postoperative day (Clavien-Dindo grade 2). Most patients were discharged on the 1st postoperative day (*n* = 292, 66.2%), 146 on the 2nd (33.1%), one on the 3rd (0.2%), two on the 4th (0.5%). Four patients (0.9%) experienced postoperative complications after 30 days from the operation that required readmission: one for an umbilical hernia requiring surgery (grade 3), two for acute pancreatitis treated conservatively (grade 2), and one for choledocholithiasis managed with endoscopic retrograde cholangiopancreatography (grade 3).

### Caseload analysis

The median caseload per operator was 18 procedures (IQR 7–30), accrued over 7 months (IQR 2–46), corresponding to a rate of one RC every 15 days (IQR 8–44). When stratified by role, surgeons performed a higher median number of procedures than trainees (28, IQR 15–43 vs. 8, IQR 3–23; Mann–Whitney test, *p* = 0.017), over a longer time span (47 months, IQR 25–59 vs. 2 months, IQR 1–5; *p* < 0.001), but with a lower procedural frequency (one RC every 45 days, IQR 22–76 vs. one RC every 8 days, IQR 6–14; *p* = 0.001).

**Fig. 2:**
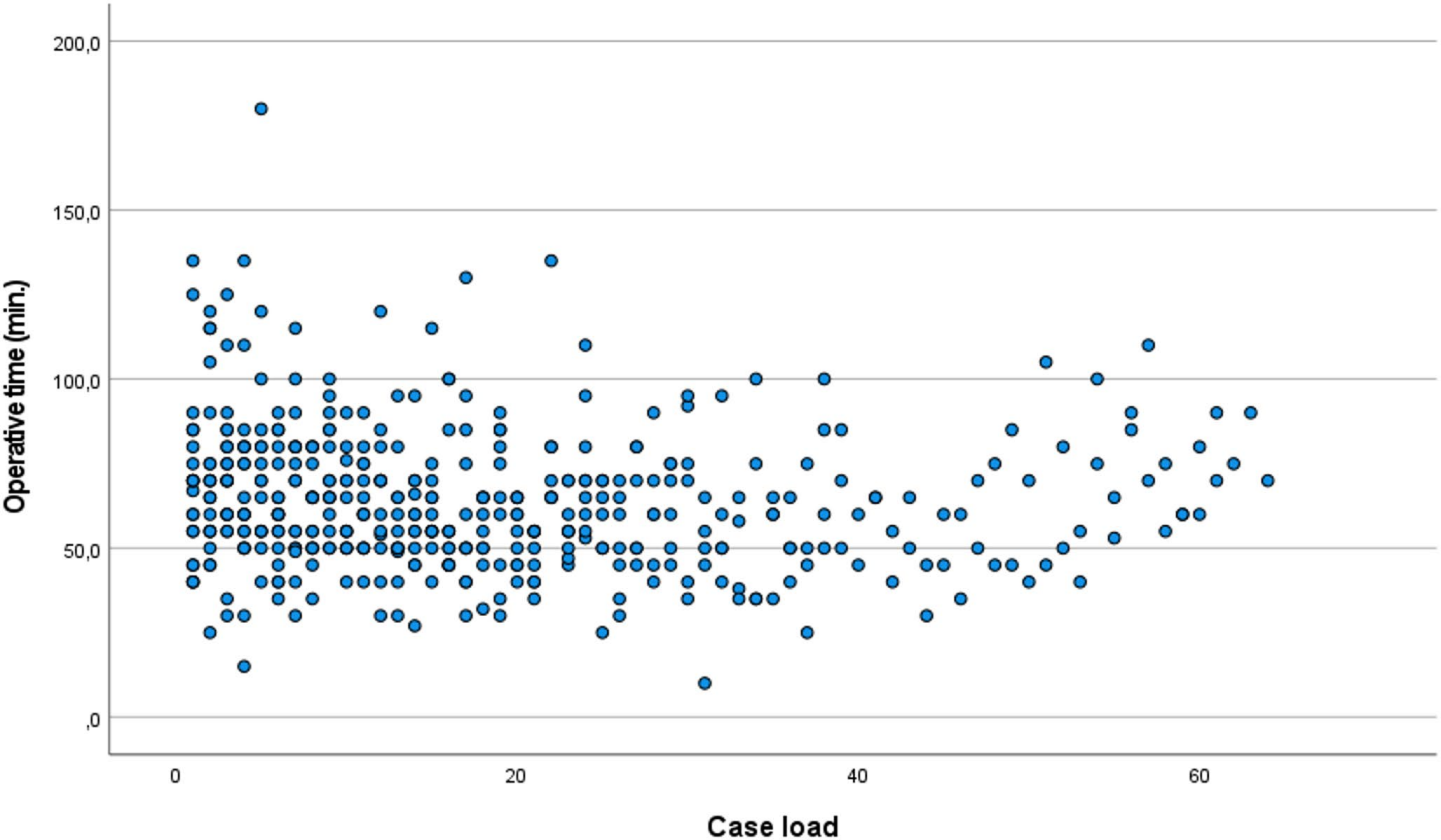
Relationship between the number of procedures performed by each surgeon and the corresponding operative times

A significant inverse correlation was observed between operative time and surgeon case load (Spearman’s *r* = − 0.145, *p* = 0.002; Fig. 2). Operative time was also significantly longer in male patients compared to females (ANOVA one-way, *p* < 0.001), when RC was performed by surgical trainees (*p* = 0.023), and in procedures requiring intraoperative adhesiolysis (*p* < 0.001). No significant differences were observed in postoperative complications or hospital readmissions according to sex, obesity, ASA grade, prior episodes of cholecystitis, previous abdominal surgery, or intraoperative adhesiolysis (Table 1). A regression analysis was conducted to assess variables potentially influencing operative time: sex, operator (surgeon vs. surgical trainee), case load, and adhesiolysis were included in the model. The model created was highly significant (*p* < 0.001). Adhesiolysis (B = 9.382; *p* < 0.001), sex (B = 7.139; *p* < 0.001), and the constant (B = 62.782; *p* < 0.001) were significantly associated with operative time. The operator was not statistically significant (B = − 3.106; *p* = 0.174), while case load showed a trend toward significance (B = − 0.132; *p* = 0.058).

**Table 1:** Baseline characteristics and univariate analysis of factors associated with operative time, postoperative complications, and readmission in robotic cholecystectomy

	Operative time (min.)	*p*	Postoperative complications (*n* = 6)	*p*	Hospital readmission (*n* = 4)	*p*
Sex:						
• Males (*n* = 159)	69 ± 22	< 0.001 ^	1 (0.6%)	0.426 °	3 (1.1%)	1.000 °
• Females (*n* = 282)	61 ± 20		5 (1.8%)		1 (0.6%)	
Obesity:						
• Absent (*n* = 285)	63 ± 21	0.144 **^**	4 (1.4%)	1.000 °	3 (1.1%)	1.000 °
• Present (*n* = 156)	66 ± 20		2 (1.3%)		1 (0.6%)	
ASA:						
• 1 (*n* = 160)	64 ± 20		2 (1.3%)		1 (0.6%)	0.072 °
• 2 (*n* = 243)	63 ± 22	0.101 **^**	2 (0.8%)	0.136 °	1 (0.4%)	
• 3 (*n* = 38)	71 ± 21		2 (5.3%)		2 (5.3%)	
Previous cholecystitis:						
• No (*n* = 413)	63 ± 21	0.142 **^**	6 (1.5%)	1.000 °	4 (1.0%)	1.000 °
• Yes (*n* = 28)	71 ± 25		0 (0%)		0 (0%)	
Previous surgery:						
• No (*n* = 258)	65 ± 23	0.272 **^**	4 (1.6%)	1.000 °	4 (1.6%)	0.145 °
• Yes (*n* = 183)	63 ± 18		2 (1.1%)		0 (0%)	
Operator:						
• Surgeon (*n* = 301)	67 ± 21	**0.023 ^**	4 (1.3%)	1.000 °	2 (0.7%)	0.595 °
• Surgical trainee (*n* = 140)	62 ± 21		2 (1.4%)		2 (1.4%)	
Adhesiolysis:						
• Yes (*n* = 144)	61 ± 18	**< 0.001 ^**	3 (1.0%)	0.720 °	1 (0.3%)	0.104 °
• No (*n* = 297)	70 ± 25		3 (2.1%)		3 (2.1%)	

*: statistical significance, ^Anova one-way; °Chi-square test; * Mann–Whitney U test

### Learning curve analysis

**Fig. 3:**
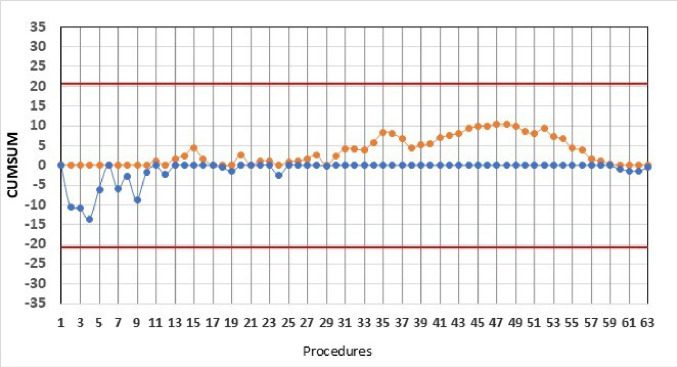
Pooled cumulative sum (CUSUM) curve. The x-axis represents the pooled chronological case sequence of the CUSUM analysis, constructed at the program level rather than at the individual operator level. The blue curve represents the cumulative sum of negative deviations from the overall mean operative time (procedures below the mean), whereas the orange curve represents the cumulative sum of positive deviations (procedures above the mean). The apparent discrepancy in the number of visible points (*n* = 63) vs. the overall CUSUM analysis (*n* = 441) is related to graphical overlap within the plotted curves, rather than to a reduced sample size

The pooled CUSUM curve demonstrated four distinct phases (Fig. 3). The initial phase was characterized by high variability, with operative times consistently above the mean and showing a progressive reduction toward it up to the 13th procedure. This was followed by a second phase, extending to the 29th procedure, during which operative times stabilized around the mean. These two phases collectively represent the learning phase. In the third phase, operative times further decreased, reaching values below the mean up to the 50th procedure. Finally, the curve exhibited an upward trend, returning toward and subsequently exceeding the mean operative time (Fig. 3). Overall, 351 patients (79.6%) were operated on during the learning phase and 90 (20.4%) thereafter, according to the pooled CUSUM analysis. Most complications (5/6; 83.3%) and all readmissions (4/4; 100%) occurred during the learning phase; however, these differences were not statistically significant (Chi-square test, *p* = 1.000 and *p* = 0.586, respectively).

**Fig. 4:**
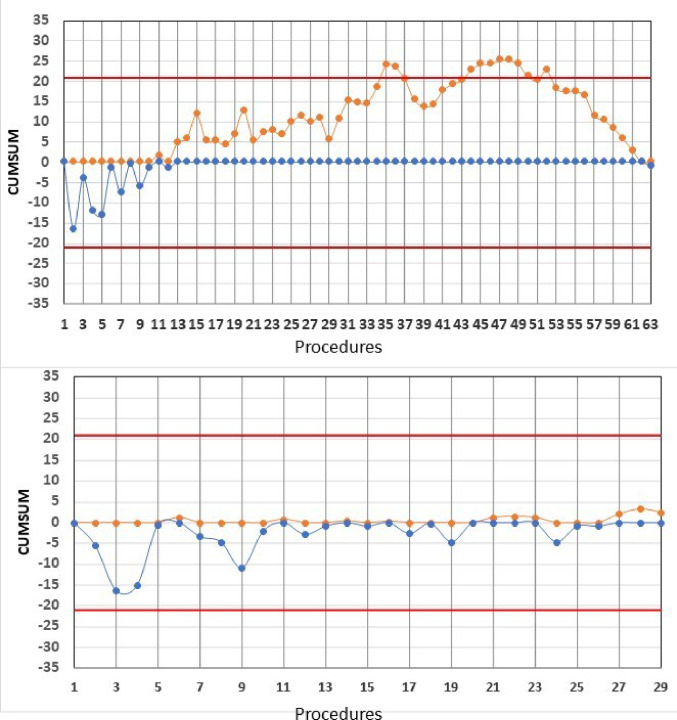
Stratified CUSUM curves according to surgeons (*top panel*) and surgical trainees (*lower panel*). The x-axes represent the pooled chronological case sequence of the CUSUM analysis, constructed at the program level rather than at the individual operator level. The blue curves represent the cumulative sum of negative deviations from the overall mean operative time (procedures below the mean), whereas the orange curves represent the cumulative sum of positive deviations (procedures above the mean). The apparent discrepancy in the number of visible points (*n* = 63) vs. the overall CUSUM analysis (*n* = 441) is related to graphical overlap within the plotted curves, rather than to a reduced sample size

A stratified CUSUM analysis according to operator type showed a comparable learning pattern between surgeons and surgical trainees. In both groups, an initial phase of variability (up to 13 cases) was followed by progressive stabilization (14–30 procedures). However, trainees showed occasional deviations from the mean beyond this point, whereas surgeons demonstrated a more consistent downward trajectory (Fig. 4).

## Discussions

This study demonstrates that RC can be safely implemented within a structured surgical training program, even in a high-volume setting involving multiple operators with no prior robotic experience. The overall complication and conversion rates were extremely low, consistent with previous studies [7, 9], while operative times were stable and lower than those reported in early series [6–9]. These findings may be explained by the high degree of procedural standardization and the cumulative experience of the surgical team, including dedicated staff and nursing personnel consistently involved in robotic procedures. Furthermore, the large sample size of our series allowed subgroup analyses across clinically relevant scenarios, including obesity, previous cholecystitis, and higher ASA scores. Favorable outcomes, in terms of low complication rates and stable operative times, were confirmed across these subgroups, supporting the feasibility and safety of this training approach in a wide range of clinical settings. Most previous studies have focused on single-surgeon or expert-led experiences [5, 8], often limiting generalizability. In contrast, the present study provides a large-scale evaluation of RC in a structured training environment, offering a more realistic representation of daily surgical practice.

The CUSUM analysis identified a clear learning pattern, characterized by an initial phase of variability (1–13 cases), followed by progressive stabilization (14–30 cases), and subsequent performance optimization (30–50 cases). Procedural stabilization was achieved after approximately 30 cases, followed by progressive performance optimization beyond this threshold, in line with previous reports [7, 13], suggesting a relatively short learning curve for RC compared with other robotic procedures [14]. The late trend observed in the CUSUM curve (> 50 cases) is likely influenced by the reduced number of observations at higher sequence levels, rather than a true decline in performance. Notably, both pooled and stratified analyses consistently identified an early stabilization threshold at approximately 13 cases, suggesting that basic technical confidence with RC can be achieved within a relatively limited number of procedures under structured supervision. The stratified CUSUM analysis provides additional insight into the learning dynamics of different operator profiles (Fig. 4). While both surgeons and trainees achieved early stabilization, the slightly increased variability observed among trainees between 14 and 30 cases (stabilization phase) possibly reflects differences in baseline laparoscopic experience (Fig. 4). However, this variability did not translate into worse clinical outcomes, reinforcing the safety of supervised training pathways.

Compared with prior studies, the present analysis confirms these findings in a larger cohort involving multiple surgeons (*n* = 22), thereby enhancing the generalizability of the results. Importantly, the learning curve observed reflects a real-world training environment, where both surgeons and trainees contributed under supervision. Despite operator heterogeneity, perioperative outcomes remained consistent across clinical subgroups, suggesting a high degree of procedural standardization. This is further supported by the absence of conversions and the low complication rates observed throughout the series. Operative time was primarily influenced by patient- and procedure-related factors, particularly adhesiolysis and male sex as already reported in the literature [15, 16], whereas operator-related variables such as training level and case load were not independently associated with operative duration, consistent with previous findings [6]. No significant differences in complication rates were observed across subgroups; however, the low number of adverse events limits the statistical power of these analyses.

This study has several limitations. First, its retrospective design may introduce selection bias, although the proportion of missing data was relatively low (3.5%). Second, some relevant variables were not available for analysis, including specific docking and undocking times, as well as detailed information on the previous laparoscopic or minimally invasive experience of the operators, which may have influenced operative performance. Third, the use of a pooled CUSUM analysis does not allow assessment of individual learning curves and may obscure inter-operator variability. However, this approach was chosen to reflect the overall performance of a structured training program rather than individual proficiency. Furthermore, limitations exist with regards to visually interpreting CUSUM inflection points as true proficiency thresholds [17, 18]. The currently presented CUSUM analysis may be useful as a descriptive visualization of program-level performance, but it does not fully support definitive conclusions about learning-curve thresholds because visually-identified inflection points provide a descriptive representation of performance trends over time but should not be interpreted as definitive or statistically-validated proficiency thresholds. Similarly, the surgeon-versus-trainee comparison should be interpreted as exploratory because the two groups differed substantially in overall caseload, duration of exposure, and procedural frequency (surgeons performed more cases over a longer period, whereas trainees performed fewer cases over a shorter period). Therefore, the conclusion that surgeons and trainees had comparable learning patterns should be interpreted cautiously. Finally, although a laparoscopic cohort was available at our institution, no direct comparison was performed. The primary aim of the present study was to evaluate the learning curve and perioperative outcomes of RC within a structured training program. A comparative analysis with LC would have required rigorous matching or adjustment for baseline differences, which was beyond the scope of this study.

## Conclusions

In a high-volume center with structured training, senior supervision, standardized technique, and appropriate case selection, RC can be implemented safely during surgical training.

These findings support the role of RC as an index procedure for training in robotic surgery, providing a reproducible and standardized platform for skill acquisition while maintaining high standards of patient safety.

## Data Availability

Data availability statement: All data are available from the Authors upon reasonable request.

## Declarations

### Originality Statement

This study provides a large, real-world evaluation of robotic cholecystectomy performed within a structured training program, including 441 consecutive cases by multiple surgeons and trainees without prior robotic experience. Results show that robotic cholecystectomy can be safely integrated into general surgery training as an entry-level robotic procedure when implemented within a structured program, high-volume setting, and appropriate case selection. The relatively short learning curve and excellent safety profile support its role as an effective platform for the acquisition of robotic skills.

## References

[1] Leal Ghezzi T, Campos Corleta O (2016) 30 Years of Robotic Surgery. World J Surg 40(10):2550–2557 27177648 10.1007/s00268-016-3543-9

[2] Tang K, Zhou W, Zhou Y, Li Y, Liao L, Dong X (2025) Robotic-assisted versus conventional/single-incision laparoscopic cholecystectomy for benign gallbladder disease: A systematic review and meta-analysis. Med (Baltim) 104(21):e42493 10.1097/MD.0000000000042493 PMC12114010 40419930

[3] Abou Assali M, Li Y, Bossie H, Neighorn C, Wu E, Mukherjee K (2025) Robotic Care Outcomes Project (ROBOCOP) for elective cholecystectomy. Surg Endosc 39(11):7262–7271 40858944 10.1007/s00464-025-12109-1 PMC12618350

[4] Coco D, Leanza S, Viola MG (2025) Single-surgeon training of 14 novice surgeons in robotic cholecystectomy: a study of 300 consecutive cases, assessing training outcomes and surgical performance. J Robot Surg 19(1):58 39891856 10.1007/s11701-024-02166-4

[5] Jayaraman S, Davies W, Schlachta CM (2009) Getting started with robotics in general surgery with cholecystectomy: the Canadian experience. Can J Surg 52(5):374–378 19865571 PMC2769095

[6] Ayabe RI, Parrish AB, Dauphine CE, Hari DM, Ozao-Choy JJ (2018) Single-site robotic cholecystectomy and robotics training: should we start in the junior years? J Surg Res 224:1–4 29506824 10.1016/j.jss.2017.07.015

[7] Vidovszky TJ, Smith W, Ghosh J, Ali MR (2006) Robotic cholecystectomy: learning curve, advantages, and limitations. J Surg Res 136(2):172–178 17059837 10.1016/j.jss.2006.03.021

[8] Juza RM, Haluck RS, Won EJ, Enomoto LM, Pauli EM, Rogers AM et al (2014) Training current and future robotic surgeons simultaneously: initial experiences with safety and efficiency. J Robot Surg 8(3):227–231 27637682 10.1007/s11701-014-0455-2

[9] Eid JJ, Jyot A, Macedo FI, Sabir M, Mittal VK (2020) Robotic Cholecystectomy Is a Safe Educational Alternative to Laparoscopic Cholecystectomy During General Surgical Training: A Pilot Study. J Surg Educ 77(5):1266–1270 32217123 10.1016/j.jsurg.2020.02.027

[10] von Elm E, Altman DG, Egger M, Pocock SJ, GÃ¸tzsche PC, Vandenbroucke JP (2007) The Strengthening the Reporting of Observational Studies in Epidemiology (STROBE) statement: guidelines for reporting observational studies. Lancet 370(9596):1453–1457 18064739 10.1016/S0140-6736(07)61602-X

[11] Dindo D, Demartines N, Clavien PA (2004) Classification of surgical complications: a new proposal with evaluation in a cohort of 6336 patients and results of a survey. Ann Surg 240(2):205–213 15273542 10.1097/01.sla.0000133083.54934.ae PMC1360123

[12] Noyez L (2009) Control charts, Cusum techniques and funnel plots. A review of methods for monitoring performance in healthcare. Interact Cardiovasc Thorac Surg 9(3):494–499 19509097 10.1510/icvts.2009.204768

[13] Liu D, Shields M, Stricklin C, Troxler C, Jarc A, Feinn R et al (2025) Early learning curve changes in objective performance indicators during robotic cholecystectomy. Front Surg 12:1679666 41141693 10.3389/fsurg.2025.1679666 PMC12550773

[14] Coco D, Leanza S (2025) Learning curve of robotic colectomy: a systematic review and meta-analysis of surgical proficiency, outcomes, and training protocols. J Robot Surg 19(1):509 40853513 10.1007/s11701-025-02648-z

[15] Bazoua G, Tilston MP (2014) Male gender impact on the outcome of laparoscopic cholecystectomy. Jsls 18(1):50–54 24680143 10.4293/108680813X13693422518830 PMC3939342

[16] Botaitis S, Polychronidis A, Pitiakoudis M, Perente S, Simopoulos C (2008) Does Gender Affect Laparoscopic Cholecystectomy? Surg Laparoscopy Endoscopy Percutaneous Techniques 18(2):157–161 10.1097/SLE.0b013e318165c899 18427334

[17] Lin PL, Zheng F, Shin M, Liu X, Oh D, D’Attilio D (2023) CUSUM learning curves: what they can and can’t tell us. Surg Endosc 37(10):7991–7999 37460815 10.1007/s00464-023-10252-1 PMC10520215

[18] Woodall WH, Rakovich G, Steiner SH (2021) An overview and critique of the use of cumulative sum methods with surgical learning curve data. Stat Med 40(6):1400–1413 33316849 10.1002/sim.8847

